# Official tenure and governance effectiveness of China's basic pension insurance system: An inverted U-shaped curve

**DOI:** 10.3389/fpubh.2022.975459

**Published:** 2022-09-20

**Authors:** Zhiguang Li, Xu Si, Wei Zhang, Zhipei Feng, Tingjing Li, Yige Guo

**Affiliations:** ^1^School of Economics and Management, Anhui University of Chinese Medicine, Hefei, China; ^2^School of Information Engineering, Anhui University of Chinese Medicine, Hefei, China; ^3^King's Business School, King's College London, London, United Kingdom

**Keywords:** basic pension insurance, official tenure, governance efficiency, DEA-Tobit, CLAD model

## Abstract

**Objectives:**

Based on incentive theory of motivation, this paper aims to estimate China's basic pension insurance's annual efficiency and inter-period efficiency changes from 2015 to 2019 and further examine the relationship between official tenure and basic pension insurance governance performance.

**Methods:**

The DEA—BCC model was used to evaluate the operating efficiency of basic pension insurance in 31 provinces of China. And four panel Tobit models were constructed to examine the heterogeneous linkages between officials' tenure and governance efficiency in different regions of China.

**Results:**

The results showed that there was an inverted U-shaped relationship between the official tenure and the governance efficiency of basic pension insurance. The younger an official was in his current position, the more apparent the inverted U-shaped relationship between the tenure of an official and the governance efficiency of basic pension insurance. We also found that localized government officials showed better governance efficiency of basic pension insurance. However, as the term of office of officials increased, the governance efficiency of non-localized officials showed a steeper negative effect.

**Conclusion:**

This study firstly reveals a significant relationship between the characteristics of officials and the operation of China's basic pension insurance system, which is a complement to the study of China's basic pension systems.

## Introduction

At the Fifth Plenary Session of the 19th CPC Central Committee, the Chinese government proposed the central task of improving the multi-level social security system. Consequently, promoting people's well-being is one of the main goals of economic and social development during the 14th Five-Year Plan period (1). Since its establishment, China's pension insurance system has had a strong capacity to pay, and its significance in maintaining social stability and promoting economic growth has been continuously reinforced (2). However, the aging of the population and unbalanced structure of the pension system impose a heavy governance burden on the fund regulator (3). Actually, the fragmentation of China's basic pension insurance system is not only characterized by geographical differences, but also by urban-rural differences (4). Therefore, the effective adjustment and allocation of the basic pension insurance system are gradually becoming an essential part of the basic security for the elderly, which is closely related to the overall situation of reform, development and stability.

There are many studies on China's basic pension systems, such as evolution of pension reform (5–7), assessments of urban employees' pension plan and new rural pension scheme (8–11), and simulation of the effect of delayed retirement on welfare of the elderly (12–15). It is noteworthy, however, that the current literature on basic pension insurance system still lacks systematic and integrated research to examine the relationship between official characteristics and governance performance. To our knowledge, the rational allocation of basic pension insurance system is related to officials' performance, and the implementation of officials will affect personal promotion (16). Therefore, officials also have governance motives, hoping that basic pension insurance system will be more efficient and it can be promoted accordingly (17). In view of theoretical knowledge of politician' incentives and behaviors for career advancement (18), we believe that there is a relationship between the characteristics of officials and the governance performance of China's basic pension insurance system. This paper aims to address three questions: (1) What is the specific relationship between official tenure and the governance effectiveness of the basic pension insurance system? (2) How does the age of officials moderate the relationship between the tenure of officials and the governance effectiveness of basic pension insurance system? and (3) How does the localization of officials moderate the relationship between the tenure of officials and the governance effectiveness of basic pension insurance system? To solve these problems, we use the data of the basic pension insurance system provinces from 2015 to 2019 as a sample to empirically examine the impact of officials' characteristics on the governance performance of China's basic pension insurance system.

The study offers some important insights into three ways. First, it uses the DEA-Tobit method and combines the sample data of the basic pension insurance system in each province of China. This study firstly reveals a significant relationship between the characteristics of officials and the operation of China's basic pension insurance system, which is a complement to the study of China's basic pension systems. Second, this paper uses the sample data related to the characteristics of officials in the regression analysis, and further confirms the empirical results obtained by the Tobit model through CLAD method. We found that there is an inverted U-shaped relationship between official tenure and governance effectiveness in the field of social security, which is a new finding in the study of official incentives and governance. Third, in the early 1980s, China established a cadre retirement system, which created institutional conditions for personnel management and realized the rejuvenation and knowledge of government officials and cadres. Meanwhile, the research finds that the current age of officials can moderate the relationship between official tenure and the governance effectiveness of basic pension insurance system, which also verifies the scientific nature of the system. Therefore, this paper also has management inspiration and guiding significance for the actual decision-making of government departments.

We conducted a literature review based on the variables and propose three hypotheses in the second part. The third part discusses the research methods and variables of the operation efficiency of China's basic pension insurance system. The fourth part makes an empirical analysis and test on the operation efficiency of pension insurance system based on the characteristics of officials. In the fifth part, we draw conclusions and suggestions on improving the operational efficiency of China's basic pension insurance system from the perspective of official characteristics in practical terms.

## Theoretical background

### Official tenure and governance effectiveness of basic pension insurance system

In the 1990s, scholars discussed the role of the political system and government governance on regional or national economic development (19). The role of governance, quality and effectiveness of regional economic growth has aroused interest (20, 21). Since then, official tenure, as an important factor affecting political incentive of motivation, has aroused many thoughts on its impact on economic growth (22). Since the reform and opening up, economic development data and tenure records of provinces across China show that the relationship between tenure and national economic development exhibits an inverted U-shaped non–linear relationship (23). A similar phenomenon also occurs in marine environmental pollution and environmental efficiency. Jiang and Li (24) confirmed that the tenure of local officials and marine environmental pollution have an inverted U-shaped non–linear relationship, and the effect of promotion incentive on the quality of the marine environment is aggravated by the tenure of local officials (24). In addition, Lu. et al. (25) also confirmed that this inverted U-shaped relationship also existed in mayors' tenure and environmental efficiency (25). Based on the above analysis, we put forward the hypothesis:

H1: There is an inverted U-shaped relationship between the official tenure and the governance efficiency of China's basic pension insurance system.

### The current age of officials and the governance effectiveness of basic pension insurance system

Visions are important to organizational effectiveness (26). In China, the age of an official reflects the ability and professional experience to a certain extent, and the knowledge and experience of local officials increase with age (27). Economic development and environmental governance are two significant indicators related to the level of social welfare. With the increase of tenure, the impact of local officials on carbon emissions showed an inverted U-shaped relationship, and younger local officials were more likely to lead to a rise in carbon emissions (28). By constructing an official competition game scenario, young officials hope to maintain a good record and upward momentum in all tenures, and tend to improve their jurisdictions' economic performance in a short time. At the same time, it is found that veteran cadres are good at overall planning. They tend to balance economic development and environmental protection (29). Therefore, we assume that the current age of officials will moderate the relationship between official tenure and the governance effectiveness of China's basic pension system. The hypotheses are developed as follows:

H2a: The younger the official is, the more significant the inverted U-shaped relationship between official tenure and the governance effectiveness of the pension system.
H2b: The older the official is, the weaker the inverted U-shaped relationship between official tenure and the governance effectiveness of basic pension insurance system.

### The localization of officials and the governance effectiveness of basic pension insurance system

In China, government officials play an indispensable role in promoting the rapid development of the regional economy (22). At the same time, China's unique cadre system has the function of promoting the diffusion of local policy innovations. Among them, government officials' cross-regional redeployment (or relocation) significantly affects local economic development and environmental protection (30). Furthermore, the diffusion method of policy innovation after officials was transferred to different places, and the reasons they were able to quickly promote their own innovation experience could be effectively explained by democratic evaluation reform and institutional merger reform (31). Hence, we hypothesize that official localization will moderate the relationship between official tenure and the governance effectiveness of basic pension system. The hypothesis is developed as follows:

H3: Official localization moderates the relationship between official tenure and the governance effectiveness of basic pension system.

## Methods

### DEA-BCC model

We use the input-oriented BCC model to calculate the technical efficiency, pure technical efficiency and scale efficiency of each decision-making unit (DMU). When the technical efficiency is equal to 1, it means that the DMU is at the technical frontier; when the technical efficiency is <1, it means that the DMU has not yet reached the production frontier, indicating that the basic pension insurance allocation efficiency has not yet reached the optimum. The BCC model can be expressed as:


$$\begin{array}{l} \min\left[\theta -\varepsilon \left(e^{T}S^{-}+e^{T}S^{+}\right)\right]=V_{TE}\\ \overset{n}{\underset{i=1}{\sum }}X_{i}\lambda_{i}-S^{-}=\theta X_{i0}\\ \overset{n}{\underset{i=1}{\sum }}Y_{i}\lambda_{i}-S^{+}=\theta Y_{i0}\\ \overset{n}{\underset{i=1}{\sum }}\lambda_{i}=1 \end{array}$$

Among them, **λ_*i*_** ≧ 0, *i*=1,2,3,…,n, *S*^+^ ≧ 0, *S*^−^ ≧ 0; θ represents the effective value of the decision-making unit, *S*^+^, *S*^−^ and *e* represent the input slack variables, the remaining variables and Non-Archimedes infinitesimals. *X* and *Y* represent the input and output combinations related to the governance performance of pension insurance system, respectively, and **λ_*i*_** represents the weight of the *i*-*th* decision-making unit.

### Tobit regression analysis

The sample used in this paper is panel data of China's provinces from 2015 to 2019. Since the efficiency evaluation result of BCC model calculation, that is, the technical efficiency of DEA (Data Envelopment Analysis) model calculation, was constrained between [0, 1], it is an indirect observation variable. When it was used as a dependent variable, an estimation bias occurred if the Ordinary Least Squares (OLS) method was used to estimate the incompleteness of the data presentation. Therefore, we used the Tobit panel regression model to analyze how the characteristics of Chinese officials affect the governance efficiency of the basic pension insurance system. The model is as follows:


(1) $$\begin{array}{l} TE_{i,t}=\alpha_{0}+\alpha_{1}Age_{i,t}+\alpha_{2}Local_{i,t}+\alpha_{3}Controls_{i,t}+\varepsilon_{i,t} \end{array}$$

(2) $$\begin{array}{l} TE_{i,t}=\alpha_{0}+\alpha_{1}Tenure_{i,t}+\alpha_{2}Tenure_{{\;}_{i,t}}^{{\;}^{2}}+\alpha_{3}Age_{i,t}\\ +\alpha_{4}Local_{i,t}+\alpha_{5}Controls_{i,t}+\varepsilon_{i,t} \end{array}$$

(3) $$\begin{array}{l} TE_{i,t}=\alpha_{0}+\alpha_{1}Tenure_{i,t}+\alpha_{2}Tenure_{i,t}^{{\;}^{2}}+\alpha_{3}Age_{i,t}\\ \times Tenure_{i,t}^{2}+\alpha_{4}Age_{i,t}+\alpha_{5}Local_{i,t}+\alpha_{6}Controls_{i,t}\\ +\varepsilon_{i,t} \end{array}$$

(4) $$\begin{array}{l} TE_{i,t}=\alpha_{0}+\alpha_{1}Tenure_{i,t}+\alpha_{2}Tenure_{i,t}^{{\;}^{2}}+\alpha_{3}Local_{i,t}\\ \times Tenure_{i,t}^{2}+\alpha_{4}Age_{i,t}+\alpha_{5}Local_{i,t}+\alpha_{6}Controls_{i,t}\\ +\varepsilon_{i,t} \end{array}$$

As shown in the above formula, the dependent variable *TE*_*i,t*_ is the technical efficiency (*TE*) of the operation of the endowment insurance system in region *i* in period *t*. α_0_ is the intercept-term. α_1_,α_2_,α_3_,α_4_,α_5_,α_6_ are the regression coefficients or coefficient matrices for the respective variables, respectively. *i* and *t* represent year *t* of province *i* respectively. ε_*it*_ represents a random variable.

### Descriptive statistics

Table 1 shows the descriptive statistics of each variable. The data shows that the average value of TE is 0.85, indicating that there is still room for improvement in the operation efficiency of the basic pension insurance system in provinces at this stage. The mean value of tenure is 4.81, suggesting that on average, it takes nearly five years for officials to be promoted or changed in each province. The mean value of age is 53.15, the minimum value is 42, and the maximum value is 60, indicating that the age of officials is closely related to their work experience and official rank status.

**Table 1:** Descriptive analysis of inputs, outputs and environmental variables.

**Type**	**Variables**	**Definition**	**Mean**	**Standard deviation**	**Minimum**	**Maximum**
Dependent variable	TE	The annual operating efficiency of basic pension insurance system estimated by the BCC model	0.85	0.14	0.51	1.00
Independent variable	Tenure	Tenure	4.81	3.28	1.00	12.00
Moderating variable	Age	Official age	53.15	4.16	42.00	60.00
	Localization	1 for the place of employment is the same province as the place of birth, 0 otherwise	0.61	0.49	0.00	1.00
Control variable	Gender	1 for males, 0 for females	0.90	0.30	0.00	1.00
	Education	0 for junior college and below; 1 for undergraduate; 2 for master; 3 for doctorate	1.44	0.56	0.00	3.00
	Experience	Whether the previous work history involves finance, financial and social security is 1; otherwise, it is 0	0.97	0.17	0.00	1.00
	Public Expenditure	Local public service expenditure/local GDP	0.28	0.11	0.15	0.63
	Social Security	Social Security and Employment Expenditure/Local Public Service Expenditure	0.14	0.04	0.08	0.27

The mean value of localization of officials is 0.61 and the mean value of gender of officials is 0.90, indicating that officials in the provinces serve as officials in their home provinces more than officials in other places, and there are more male officials than female officials. The average value of education is 1.44, showing that there are a large number of officials with a bachelor's degree or above, and a good level of education also helps officials to be promoted to a certain extent. In addition, the previous work history of the vast majority of officials involves finance and social security. The standard deviations of public expenditure and social security are both around 30% of the mean, suggesting that there is little difference in local government spending in different public areas.

#### Dependent variables

*Technical efficiency (TE)*. According to the input and output variables determined by the efficiency evaluation index system, we estimate the BCC model, and obtain the annual efficiency value of the governance performance of the basic pension insurance system in each province, as well as the technical changes and efficiency changes. We therefore adopt the technical efficiency to measure the governance effectiveness of China's basic pension insurance system.

#### Independent variables

*Tenure*. The article uses the provincial social security bureau director or the deputy director in charge of the Human Resources and Social Security Department to measure the tenure, which is conducive to studying the impact of officials' characteristics on the governance performance of China's basic pension insurance system.

#### Moderating variables

The moderating variables of this paper are as follows: (1) *Current age of officials*. We use the age of the director of the provincial social security bureau or the deputy director of the department of human resources and social security to measure the age of the official. (2) *Localization of officials*. We use 0 to indicate that the place where officials take office is different from the place of birth, and 1 to suggest that the place where officials take office is the same as the place of birth.

#### Control variables

We control the following variables: (1) *Gender of officials (Gender)*. We use 0 for female and 1 for male. (2) *The education of officials (Education)*. We use 0 for junior college and below, 1 for undergraduate, 2 for master, and 3 for doctorate. (3) *Official work experience (Experience)*. We use 0 to represent that the official's previous work history does not involve finance and social security, and 1 to mean that the previous work history involves finance and social security. (4) *The scale of government public expenditure (Public Expenditure)*. The level of regional public financial expenditure is measured by the ratio of local public financial expenditure to provincial GDP. (5) *Social security expenditure scale (Social Security)*. The level of social security expenditure is measured by the proportion of local social security and employment expenditure to local public service expenditure.

### Data sources

This paper aims to reveal the impact of the characteristics of officials in China's provinces on the governance efficiency of China's pension insurance system from 2015 to 2019. The data of the inputs, outputs, government public expenditure and social security expenditure in the efficiency evaluation indicators come from 2016 to 2020 *China Statistical Yearbook* and the statistical yearbooks of individual provinces, covering the relevant data of the basic pension insurance system in 31 provinces across China. The information on the characteristics of officials comes from the leaders of the department disclosed in the government affairs disclosure section on the official website of the Human Resources and Social Security Bureau or related news reported by the media.

### Correlation analysis

**Figure 1:**
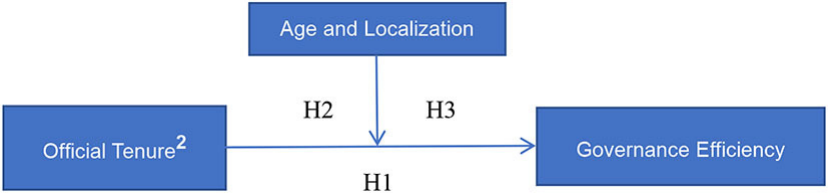
The research model.

Since the DEA-BCC model requires a strong correlation between outputs and inputs, it is necessary to analyze the correlation between variables. The results of the Pearson correlation analysis between the variables are shown in Table 2. There is no significant correlation between tenure and the governance effectiveness of basic pension insurance system. There may be a non–linear relationship between tenure and the governance effectiveness of the pension system, but not a linear correlation. At the same time, to test the multi-collinearity problem, we consequently calculated the variance inflation factors (VIFs), and found that the maximum value of VIF was 1.50 and the average value was 1.26, which effectively indicated that there was no apparent multicollinearity relationship between the variables selected in this paper. Figure 1 provides a summary of our conceptual framework and shows the hypotheses to be tested in the empirical analysis.

**Table 2:** Pearson correlation analysis results.

**Variables**	**TE**	**Tenure**	**Age**	**Localization**	**Gender**	**Education**	**Experience**	**Public**	**Social**	**VIF**
								**expenditure**	**security**	
TE	1									–
Tenure	0.0472	1								1.15
Age	0.0329	0.1293	1							1.38
Localization	0.2559^*^	0.0351	0.1875	1						1.23
Gender	0.1488	0.2255^*^	0.294^**^	−0.1982^*^	1					1.50
Education	0.1302	0.0075	−0.2733^**^	−0.2163^*^	0.265^*^	1				1.36
Experience	0.0063	0.1513	−0.0786	0.2199^*^	−0.0586	0.1398	1			1.16
Public expenditure	−0.0887	−0.0972	0.2033^*^	0.171	0.1137	−0.0774	0.1268	1		1.14
Social security	0.4034^**^	−0.0449	0.0376	−0.0562	0.2485^*^	0.2369^*^	−0.0233	−0.0584	1	1.13

* Indicates significant p values at the 5%.

** Indicates significant p values at the 1%.

^***^Indicates significant p values at the 0.1%.

## Empirical results

### Hypothesis testing and analysis

Both the mixed Tobit regression model and random effects panel Tobit regression model are typical Tobit regression models for panel data. In addition, the results of the likelihood ratio test (LR test) can be effective in helping to determine which model should be used. The results of the LR test for this model show that the null hypothesis was rejected (*p* value was 0.000), so it was considered that there was an individual effect, and a panel Tobit regression with random effects could be used. Therefore, we conducted a Tobit regression analysis on the model. The results of the regression analysis of the official characteristics and governance performance of the Chinese pension system are shown in Table 3.

**Table 3:** Results of mediation and moderated mediation regression analyses.

**Variables**	**Model 1**	**Model 2**	**Model 3**	**Model 4**
Tenure		0.0222445^**^	0.0284933^**^	0.0206107^*^
Tenure^2^		−0.0018264^*^	−0.0131436^***^	−0.0028526^***^
Age	−0.0063902^*^	−0.0092824^**^	−0.0142232^***^	−0.0099972^**^
Localization	0.077064^**^	0.0799235^**^	0.0789798^**^	0.0535124
Tenure^2^ × age			0.0001976^**^	
Tenure^2^ × localization				0.0016955^**^
Gender	0.1675998^***^	0.1641058^***^	0.1568517^***^	0.1632861^***^
Education	0.0159188	−0.0007987	0.0064307	0.0048638
Experience	0.0457511	0.0431129	−0.0129012	0.0267285
Public expenditure	−0.1228898	−0.1317106	−0.1428199	−0.1515982
Social security	0.2706763	0.3643664	0.5907052	0.7095143
_cons	0.9474027	1.046152	1.325041	1.072054
LR test of chibar^2^	43.81	42.76	34.14	41.59
Prob ≥ chibar^2^	0	0	0	0

* Indicates significant p values at the 5%.

** Indicates significant p values at the 1%.

*** Indicates significant p values at the 0.1%.

As shown in Table 3, Model 1 is the regression result of control variables and moderator variables on the governance efficiency of China's basic pension insurance system. Model 2 is the regression result after adding explanatory variables. Model 3 and Model 4 are the regression results after adding interactive items of age and localization respectively. The data show that these models are statistically significant. Among the moderating variables, age has a significantly negative correlation, and localization also has a significantly positive correlation in model 1, indicating that the younger the officials serving and when they are the locals, the higher the governance efficiency of the fund. The results in Model 2 show that the regression coefficients of tenure and governance effectiveness are both significantly positive (r = 0.0222445, *p* < 0.05), but their square terms show a significant negative relationship (r = −0.0018264, *P* < 0.1), indicating that the relationship between the tenure and the governance effectiveness of the basic pension insurance system presents an inverted U-shaped relationship.

**Figure 2:**
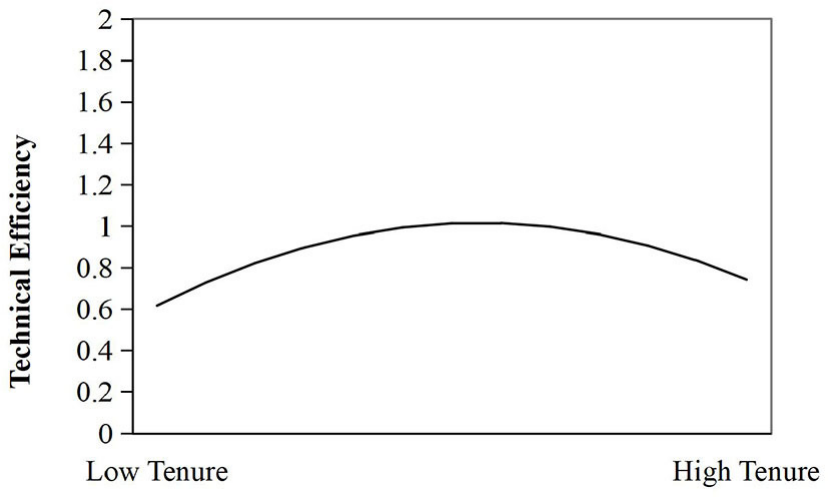
The inverted U-shaped curve between Tenure and TE.

According to the results of Model 2, the value of the explanatory variable at the inflection point of the curve is −β_1_/2β_2_=6.09, indicating that when tenure is in the 6.09th year, the governance efficiency of the basic pension insurance system will reach the optimum. Therefore, H1 is supported. The inverted U-shaped curve between tenure and fund governance effectiveness is shown in Figure 2.

**Figure 3:**
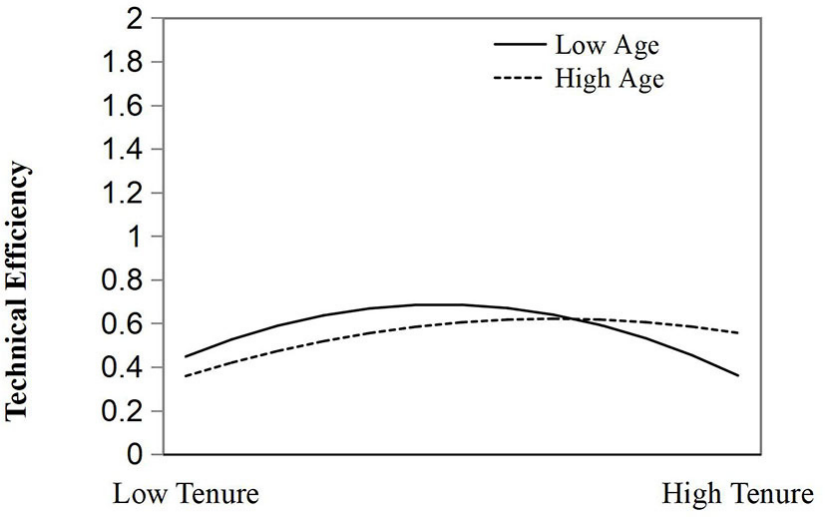
The moderating effect of official age.

H2a and H2b discuss the moderating effect of age on the relationship between tenure and fund governance effectiveness, respectively. In Model 3, the regression results show that the coefficient of interaction between the square term of tenure and age is significantly positive (r = 0.0001976, *p* < 0.01). Therefore, both H2a and H2b are supported. Specifically, when the official is younger, the work enthusiasm is higher, and the inverted U-shaped relationship between tenure and the governance efficiency is more significant, which confirms the moderating effect of age on the relationship between tenure and fund governance effectiveness. However, as the age of official increases, once young officials reach a certain age and have not been promoted, this relationship will reverse, showing a trend of declining governance efficiency. Corresponding older government officials have similar problems, but the declining trend is smaller than the negative effect of young officials. The moderating effect is shown in Figure 3.

**Figure 4:**
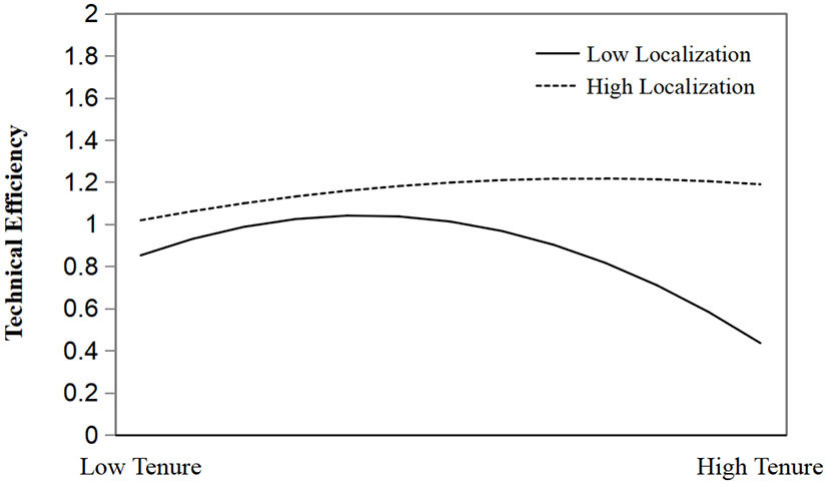
The moderating effect of localization.

In addition, in Model 4, the regression results show that the coefficient of the interaction term of the square term of tenure × localization and the governance efficiency of basic pension insurance system is significantly positive (r = 0.0016955, *p* < 0.01), which confirms the interaction effect, and H3 is supported. This shows that compared with non-localized government officials, localized government officials show better governance efficiency of basic pension insurance system. However, with the increase of official tenure, the governance effectiveness of non-localized government officials shows a steeper negative effect. This may be due to the fact that local officials have richer network resources and maintain a certain degree of emotional dependence on the place of birth, hoping to leave a better reputation. On the other hand, non-localized officials are limited by their own resources and lack emotional attachment. If they fail to be promoted during their expected tenure, there will be a huge psychological gap, which is more likely to lead to the phenomenon of sloth administration. The adjustment effect is shown in Figure 4.

### Robustness analysis

According to research of Wilhelm and Pullenayegum (32, 33), the estimation results of the censored least absolute deviations (CLAD) and Tobit models should be similar. Therefore, we use CLAD as a robustness test for the Tobit model. To further confirm the empirical results obtained by the Tobit model, we adopt a more robust CLAD estimation method. The results of using the CLAD model to regress the tenure and the governance performance of China's pension insurance system are listed in Table 4.

**Table 4:** Results of regression analysis based on CLAD Model.

**Variables**	**Model 1**	**Model 2**	**Model 3**	**Model 4**
Tenure		0.0360932^***^	0.0412751^***^	0.033619^***^
Tenure^2^		−0.003562^***^	−0.0141469^***^	−0.0045271^***^
Age	−0.0010828^***^	0.0020477^***^	−0.0064169^***^	−0.0048568^***^
Localization	0.1055972^***^	0.1092921^***^	0.0687195^***^	0.0476373^***^
Tenure^2^ × age			0.0001862^***^	
Tenure^2^ × localization				0.0019585^***^
Gender	0.1085609^**^	0.0950154^***^	0.1366039^***^	0.1338566^**^
Education	−0.001022^***^	−0.0072942^***^	0.0136494^***^	−0.0367022^**^
Public expenditure	−0.3296014^*^	−0.3240177^**^	−0.1949042^**^	−0.2370363^**^
Social security	1.124524	1.048904	0.6551612	1.024752
_cons	0.5231172	0.5231172	0.9443152	0.9063769

* Indicates significant p values at the 5%.

** Indicates significant p values at the 1%.

*** Indicates significant p values at the 0.1%.

By comparing the regression results of the Tobit method and the CLAD method, it is found that there is a certain difference in the coefficient estimates of the variables, and the standard error of CLAD is smaller than that of Tobit. Regarding the impact of tenure on governance efficiency, there is no essential difference between CLAD and Tobit in the level of variable significance, so the regression results obtained in this paper can be considered robust.

## Conclusions and implications

In order to more effectively explore the governance performance of China's basic pension insurance system based on the characteristics of officials, we use the DEA-BCC model, and take the fund income and the number of insured as input indicators, and the fund expenditure and the number of recipients as the output indicators, further estimate changes in the annual and inter-period efficiency of the basic pension insurance system in China's provinces from 2015 to 2019. At the same time, by constructing Tobit regression models, the normality and homoscedasticity of the disturbance term in the Tobit model are tested, and the influence of officials' characteristics on the governance efficiency of basic pension insurance system is examined. Finally, we further validate the empirical results of the Tobit model by means of CLAD. The results are as follows: (1) We found that the operational efficiency of China's basic pension insurance system needs to be improved. After further research, there is an inverted U-shaped relationship between the tenure of officials and the governance effectiveness of China's basic pension insurance system. In other words, the newly appointed officials showed better governance effectiveness of of China's basic pension insurance system in the early tenure, but when the official's tenure exceeds 6.09 years, the governance efficiency of China's basic pension insurance system will decrease accordingly. (2) The current age of officials will moderate the relationship between official tenure and the governance effectiveness of China's basic pension insurance system. The younger the current age of officials, the more steeper the inverted U-shaped relationship between official tenure and the governance effectiveness because of officials' self-efficacy and negative emotional responses. (3) The localization of officials will moderate the relationship between official tenure and the governance effectiveness of China's basic pension insurance system. For those officials whose current employment and birthplace are in the same province, the positive relationship between officer tenure and fund governance efficiency becomes more pronounced in the early stages, and the negative relationship flattens out in the later stages. While for those officials whose employment and birthplace are not in the same province, the positive relationship between officer tenure and fund governance effectiveness is relatively stable in the early stages, but the negative relationship becomes steeper in the later stages, when they are not promoted.

To sum up, in order to further optimize the allocation of fund-related resources and effectively improve governance efficiency, we put forward the following suggestions: Firstly, this study has provided a deeper insight into the government, which needs to pay attention to the psychological state of officials in a timely manner, and conduct appropriate emotional counseling. Every official will gradually have his own professional expectations as his tenure grows, hoping to feel fairness and justice from the government's response. Secondly, the results of this paper support the view that governments should avoid age discrimination and ensure that all officials have access to experience, training and promotion. For government officials, age is not only a natural physiological indicator, but also a comprehensive social indicator that reflects professional identity. To a certain extent, young officials have a strong pioneering and progressiveness, which is conducive to accepting new things and confirms the government's new requirements for officials in the era of rapid development. Thirdly, it is necessary for the government to encourage local officials to make efforts to create a government culture that keeps pace with the times and makes everyone progress. Localization officials have a thorough understanding of the actual situation of the province, and have a strong awareness of resource allocation and organizational coordination, which is conducive to the improvement of the governance efficiency of basic pension insurance. Therefore, localization factors can be taken into account in job transfer and personnel arrangement to maximize the motivation of officials.

## Data availability statement

The original contributions presented in the study are included in the article/supplementary material, further inquiries can be directed to the corresponding author.

## Author contributions

ZL contributed to conceptualization, methodology, validation, investigation, analysis, writing of the study, administration, and funding acquisition. WZ and ZF supervised the study and contributed to conceptualization, methodology, investigation, writing and editing, and visualization. TL and YG contributed to methodology and review and editing. XS contributed to data validation and review of the study. All authors read and approved the final manuscript.

## Funding

This study was funded by Anhui Province Federation of Social Sciences under Grant No. 2021CX093; Nature Science Foundation of Anhui Provincial Education Department under Grant No. KJ2021A0583; Industry-University-Research Cooperation Program of the Chinese Ministry of Education Grant No. 202102469005 and No. 202102126064; Youth Talent Program of Anhui University of Chinese Medicine Grant No. 2021qnyc12.

## Conflict of interest

The authors declare that the research was conducted in the absence of any commercial or financial relationships that could be construed as a potential conflict of interest.

## Publisher's note

All claims expressed in this article are solely those of the authors and do not necessarily represent those of their affiliated organizations, or those of the publisher, the editors and the reviewers. Any product that may be evaluated in this article, or claim that may be made by its manufacturer, is not guaranteed or endorsed by the publisher.
